# The Effect of Hydration on Urine Color Objectively Evaluated in CIE L^*^a^*^b^*^ Color Space

**DOI:** 10.3389/fnut.2020.576974

**Published:** 2020-10-26

**Authors:** Rebekah Belasco, Tory Edwards, A. J. Munoz, Vernon Rayo, Michael J. Buono

**Affiliations:** School of Exercise and Nutritional Sciences, San Diego State University, San Diego, CA, United States

**Keywords:** urine color, urine osmolality, dehydration, color space, CIE L^*^a^*^b^*^

## Abstract

Urine color has been shown to be a viable marker of hydration status in healthy adults. Traditionally, urine color has been measured using a subjective color scale. In recent years, tristimulus colorimetry developed by the International Commission on Illumination (CIE L^*^a^*^b^*^) has been widely adopted as the reference method for color analysis. In the L^*^a^*^b^*^ color space, L^*^ indicates lightness ranging from 100 (white) to 0 (black), while a^*^ and b^*^ indicate chromaticity. a^*^ and b^*^ are color directions: –a^*^ is the green axis, +a^*^ is the red axis, –b^*^ is the blue axis, and +b^*^ is the yellow axis. The L^*^a^*^b^*^ color space model is only accurately represented in three-dimensional space. Considering the above, the purpose of the current study was to evaluate urine color during different hydration states, with the results expressed in CIE L^*^a^*^b^*^ color space. The study included 28 healthy participants (22 males and 6 females) ranging between the age of 20 and 67 years (28.6 ± 11.3 years). One hundred and fifty-one urine samples were collected from the subjects in various stages of hydration, including morning samples after 7–15 h of water deprivation. Osmolality and CIE L^*^a^*^b^*^ parameters were measured in each sample. As the urine osmolality increased, a significant linear increase in b^*^ values was observed as the samples became more pronouncedly yellow (τ_b_ = 0.708). An increase in dehydration resulted in darker and significantly more yellow urine, as L^*^ values decreased in lightness and b^*^ values increased along the blue–yellow axis. However, as dehydration increased, a notable polynomial trend in color along the green–red axis was observed as a^*^ values initially decreased, indicating a green hue in slightly dehydrated urine, and then increased as urine became more concentrated and thus more dehydrated. It was determined that 74% of the variance seen in urine osmolality was due to CIE L^*^a^*^b^*^ variables. This newfound knowledge about urine color change along with the presented regression model for predicting urine osmolality provides a more detailed and objective perspective on the effect of hydration on urine color, which to our knowledge has not been previously researched.

## Introduction

Urine color has been shown to be a viable marker of hydration status in healthy adults (1, 2). This is evidenced by the significant correlations (*r* > 0.70) of urine color with several established physiological measures of whole-body hydration, including urine osmolality and urine-specific gravity (1, 3–5). Urine color is dictated by the concentration of urochrome, a yellow-pigmented waste by-product of hemoglobin catabolism (1, 6, 7). During whole-body dehydration, urochrome concentration increases as antidiuresis is stimulated *via* increased arginine vasopressin production, thus increasing water reabsorption from the collecting duct of the nephron. This physiological response to dehydration results in darker and more concentrated urine (3, 4).

Traditionally, the assessment of hydration through urine color analysis has been performed with a subjective eight-point color scale (1, 3–5). This technique involves visual color matching of a collected urine sample to a stepwise color chart, resulting in an assigned value ranging from 1 (pale yellow) to 8 (dark greenish brown) (3, 4). Color assessments based on visual comparisons to a set of stepwise color samples, also referred to as color-order systems, are known to have several methodological limitations. These include the following: (1) variability of color vision in different individuals, (2) change of color vision with age, (3) potential color blindness in individuals, (4) difficulty in standardization of illumination conditions, and (5) difficulty in standardization of reference color charts (8–10). Furthermore, evidence of the eight-point color scale's success in determining hydration status in older populations is inconclusive (11, 12).

Although the eight-point color scale has proven to be a practical tool for convenient assessment of hydration status in healthy adults (1, 3–5), it lacks the capability to be used as a universally accurate tool for objective quantification of urine color.

CIE L^*^a^*^b^*^ is a system of objective tristimulus colorimetry developed by the International Commission on Illumination (i.e., Commission Internationale de l'Eclairage, CIE) in which three different characteristics constitute a color's position within a three-dimensional color space. CIE L^*^a^*^b^*^ analysis describes all colors visible to the human eye and was created to serve as a device-independent reference model. This system has been used to perform objective analysis of color and color movement in several different scientific fields, ranging from food science to medicine. Previous research has used CIE L^*^a^*^b^*^ to identify photosynthetic pigmentation in olive oils, to create ranges of human gingival color, and to visualize body movement captured by remote photoplethysmography (13–15). Furthermore, the CIE L^*^a^*^b^*^ color space has been widely adopted as a standard for color assessment and quantification since its conception in 1976 (10, 14, 16).

**Figure 1:**
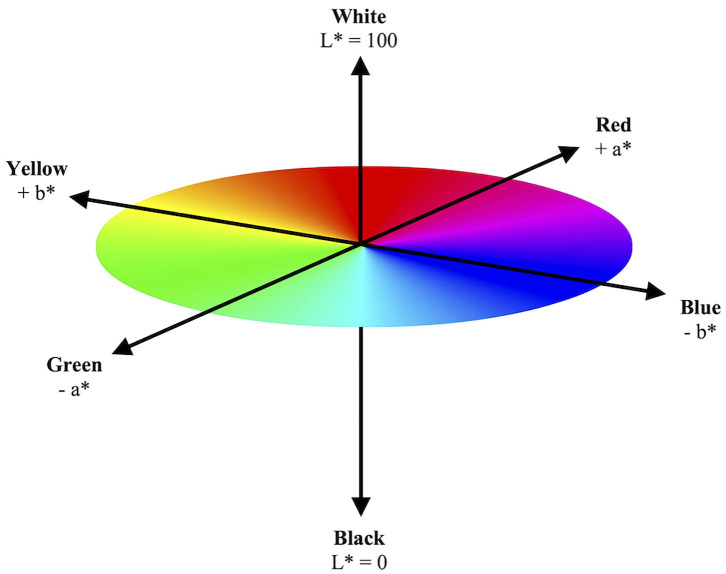
Model illustration of three-dimensional CIE L*a*b* color space. The CIE L*a*b* color space illustrates a color's objective lightness and chromaticity. The L* value measures lightness from black to white. The a* and b* values measure chromaticity on the green–red spectrum and blue–yellow spectrum, respectively.

In the L^*^a^*^b^*^ color space, L^*^ indicates lightness ranging from 100 (white) to 0 (black), while a^*^ and b^*^ indicate chromaticity or the quality of a color independent of its luminance. a^*^ and b^*^ are color directions: –a^*^ is the green axis, +a^*^ is the red axis, –b^*^ is the blue axis, and +b^*^ is the yellow axis. As the L^*^a^*^b^*^ color space is a three-dimensional model, it can only be accurately represented in a three-dimensional space (Figure 1). These three parameters are measured *via* spectral analysis of each wavelength within the visible color spectrum by a spectrophotometer.

Considering the above, the purpose of the current study was to evaluate urine color during dehydration, with the results expressed in CIE L^*^a^*^b^*^ color space parameters. As such, this study was conducted not to replace any current methods of urine analysis, but rather to provide a more objective perspective on the effect of hydration on urine color. Such data, which to our knowledge has not been previously published, would both advance our understanding about objective measurement of urine color and aid in the quantification of urine color during various stages of hydration in humans.

## Materials and Methods

A cross-sectional study was conducted to examine the relationship between hydration status and urine color when visualized in the CIE L^*^a^*^b^*^ color space. The subjects for this study were 28 healthy volunteers (22 males and 6 females) ranging between the age of 20 and 67 years (28.6 ± 11.3 years). Prior to data collection, all subjects read and signed an informed consent approved by the San Diego State University Institutional Review Board. One hundred and fifty-one spot urine samples were collected from the subjects in various stages of hydration, including morning samples after 7–15 h of water deprivation. Diet and vitamin intake were not controlled. Menstrual cycle status of the female participants was not determined.

Participants arrived at the laboratory in the morning following an overnight period of water deprivation and provided a urine sample. Participants were then instructed to rehydrate with 1 to 3 L of water over a period of 4 h. Urine samples were collected approximately once per hour during the rehydration period, thus providing samples at various stages of hydration. Urine samples were collected in plastic containers by the subject and then immediately analyzed upon retrieval. Subjective urine color was determined by two investigators using the eight-point urine color chart (3, 4). Urine osmolality was measured in duplicate using a Wescor Model 5500 vapor pressure osmometer (Logan, UT). Urine color was measured using a Hunter Lab Vista spectrophotometer (Reston, VA), and the results were expressed in CIE L^*^a^*^b^*^ color space parameters.

Statistical analyses were performed using IBM SPSS Statistics, version 26.0. Two-tailed Kendall's Tau-b correlation analyses were performed to determine the strength of the relationship between urine osmolality and the individual CIE L^*^a^*^b^*^ values. Standardized beta coefficient values were also calculated for the individual CIE L^*^a^*^b^*^ components to determine the predictor variable that was most strongly related to urine osmolality. A hierarchal multiple regression model was then constructed to determine the strength of the relationship between the predictor variables of urine color, as expressed in CIE L^*^a^*^b^*^ color space, and the criterion variable of urine osmolality. The hierarchy of predictor variables when used in the construction of the multiple regression model was based on the individual beta coefficient values. The alpha level for significance was set to α = 0.05 *a priori*.

## Results

**Figure 2:**
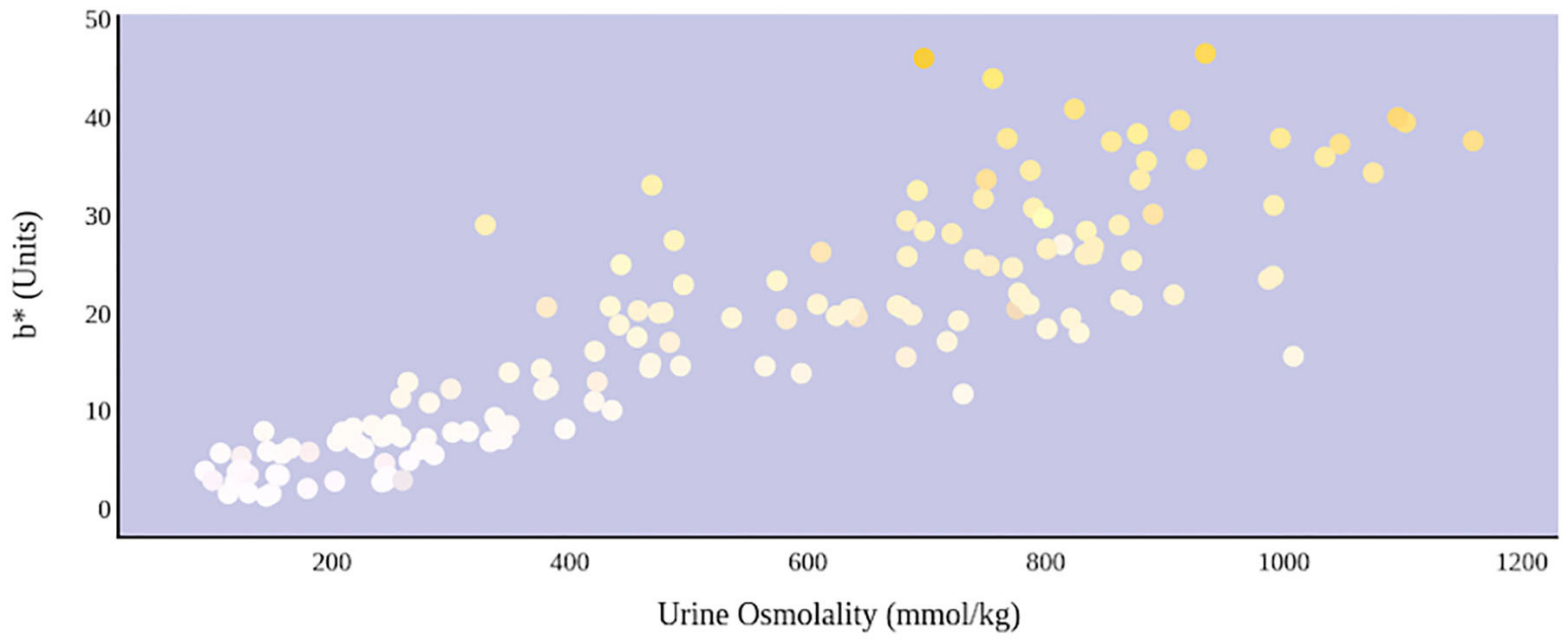
An increase in urine osmolality is significantly correlated with an increase in yellow urine color. A more dehydrated sample, as determined by urine osmolality readings, was measured as having a higher b* value. This indicates that an increase in dehydration is significantly correlated with increased yellow color in urine.

**Figure 3:**
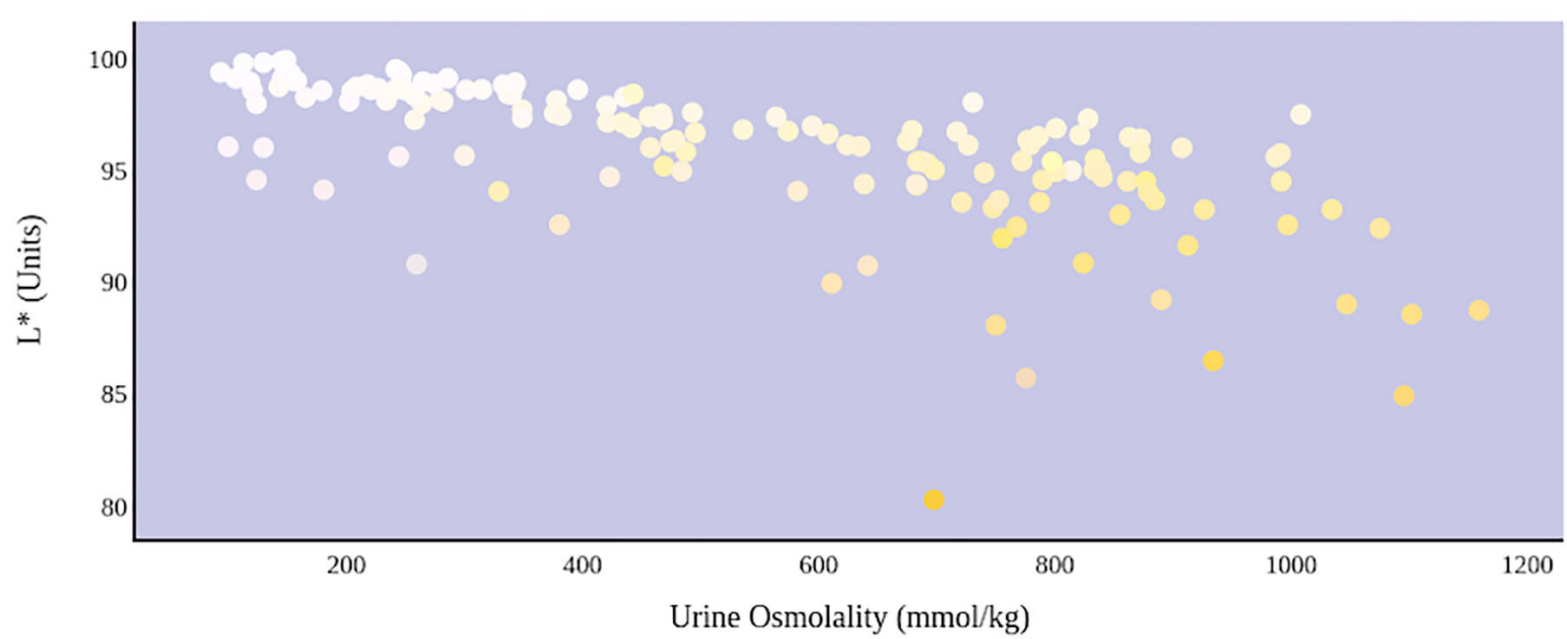
An increase in urine osmolality is significantly correlated with a decrease in urine sample lightness. A more dehydrated sample, as determined by urine osmolality readings, was measured as having a lower L* value. This indicates that an increase in dehydration is significantly correlated with darker urine color.

**Figure 4:**
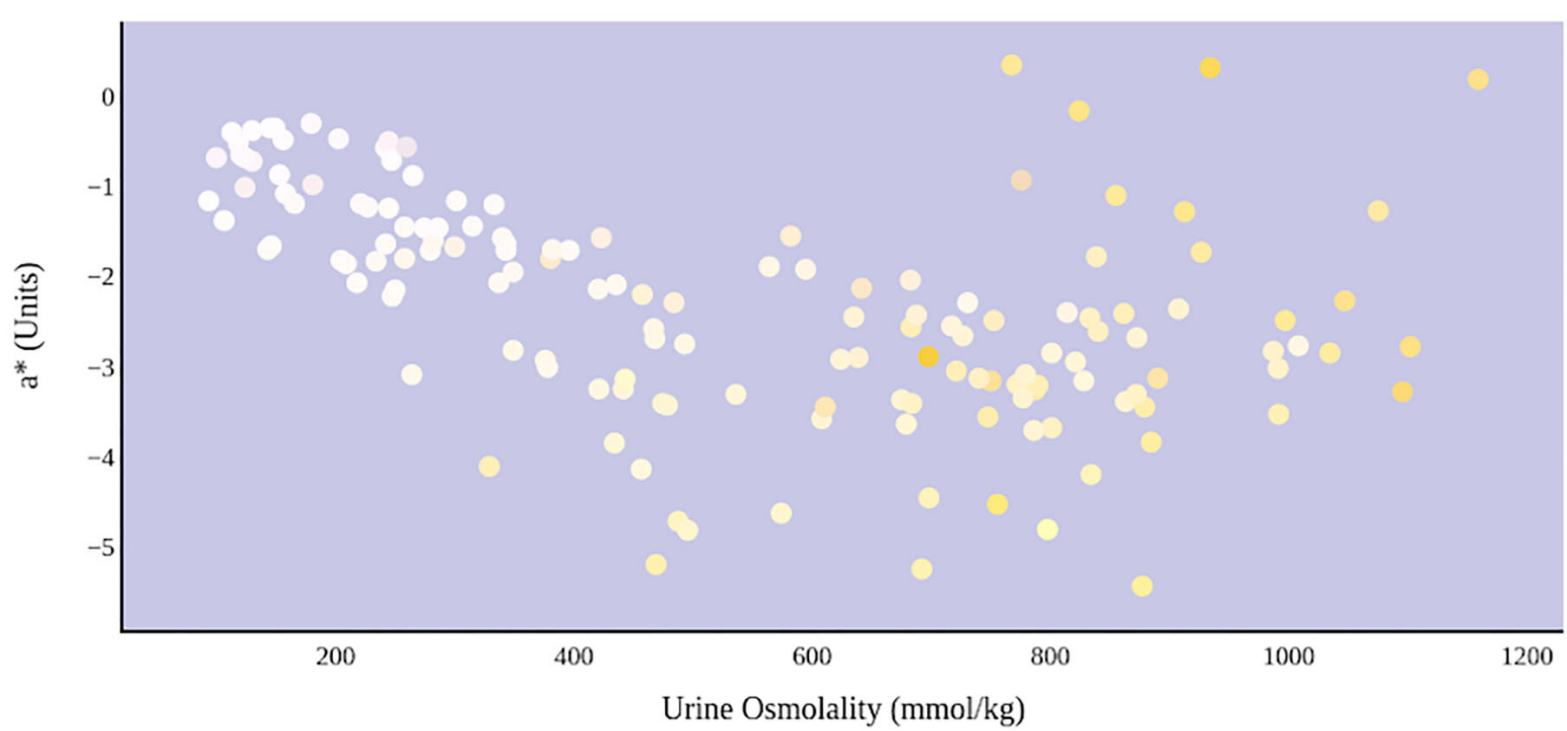
An increase in urine osmolality is significantly correlated with the movement of urine color on the green–red axis. An initial increase in dehydration, as determined by urine osmolality readings, was accompanied by a decrease in a* value. At a urine osmolality reading of ~600 mmol/kg, the a* value began to increase. This indicates a parabolic relationship between hydration status and the value of green–red chromaticity in urine.

**Figure 5:**
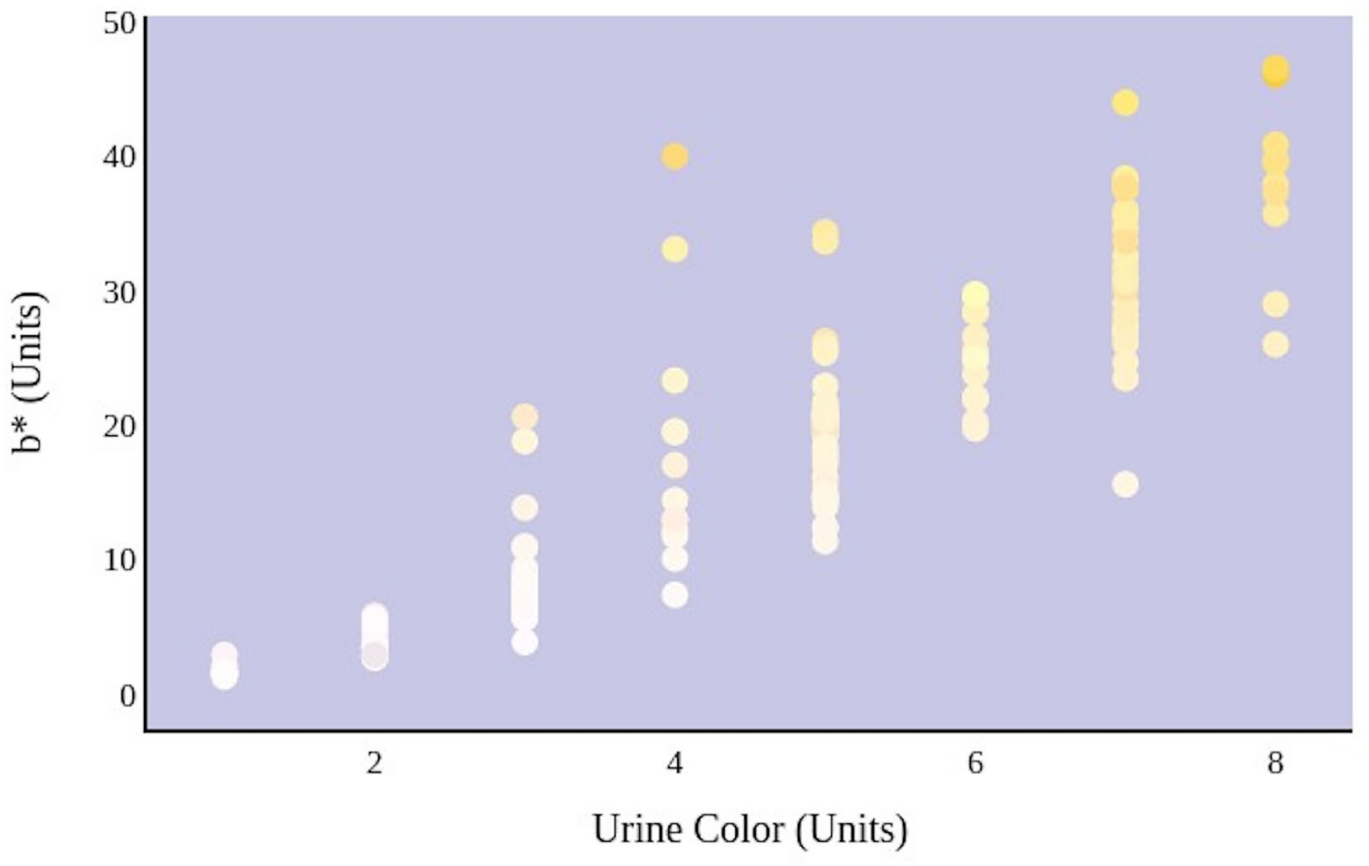
An increase in urine color score as determined by an eight-point color scale is significantly correlated with the movement of urine color on the blue–yellow axis.

A greater urine osmolality reading indicated higher concentration of urine solutes per kilogram of water and, thus, represented a more dehydrated urine sample. The urine osmolality was ~50–200 mmol/kg following water consumption and over 1,000 mmol/kg following overnight water deprivation. As the urine osmolality of the collected samples increased, a significant linear increase in b^*^ values was observed as the samples became more pronouncedly yellow (τ_b_ = 0.708) (Figure 2). An increase in urine osmolality was also accompanied by a significant linear decrease in L^*^ values as the samples became darker (τ_b_ = −0.567) (Figure 3). The relationship between urine osmolality and a^*^ values was determined to be parabolic, with an initial decrease followed by an increase in a^*^ values as urine osmolality increased (τ_b_ = −0.375) (Figure 4). Notably, a significant relationship existed between the b^*^ value of the CIE L^*^a^*^b^*^ color space and the eight-point urine color score as an increasing color chart score was associated with a higher b^*^ value (τ_b_ = 0.805) (Figure 5). The mean, standard deviation, and two-tailed Kendall's Tau-b correlations for CIE L^*^a^*^b^*^, urine osmolality, and urine color data are presented in Table 1.

**Table 1:** Means, standard deviations, and two-tailed Kendall's Tau-b correlation coefficients for CIE L*a*b*, urine osmolality, and urine color data.

**Variable**	***M***	**SD**	**U^†^_OSM_ (mmol/kg)**	${\text{U}}_{\text{COL}}^{\text{‡}}$	L^*^	a^*^	b^*^
${\text{U}}_{\text{Osm}}^{\text{†}}$ (mmol/kg)	538	291	–				
${\text{U}}_{\text{Col}}^{\text{‡}}$	4.62	1.94	0.704^*^	–			
L^*^	95.8	3.4	−0.567^*^	−0.622^*^	–		
a^*^	−2.2	1.5	−0.375^*^	−0.440^*^	0.262^*^	–	
b^*^	18.5	12.2	0.708^*^	0.805^*^	−0.696^*^	−0.506^*^	–

* p < 0.05.

† *Urine osmolality abbreviated to U_Osm_*.

‡ *Urine color abbreviated to U_Col_*.

**Figure 6:**
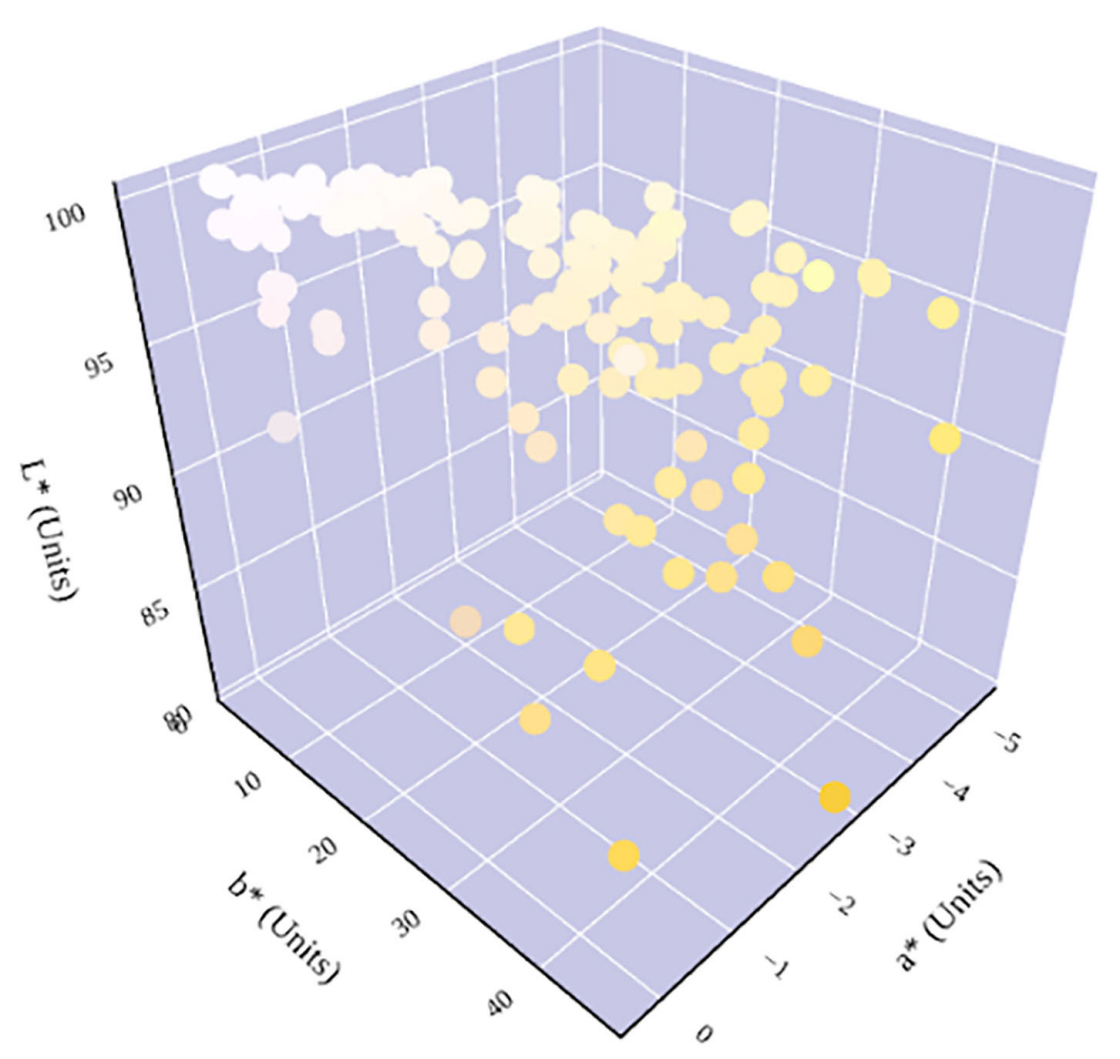
Hydration status significantly determines urine color in the three-dimensional CIE L*a*b* color space. An increase in dehydration results in an increase in b* and a decrease in L*. The relationship between hydration status and a* is parabolic. A notable shift in a* trend occurs at ~600 mmol/kg, which has previously been identified as being in the cutoff range for distinguishing between whole-body euhydration and dehydration (4, 17–19).

**Figure 7:**
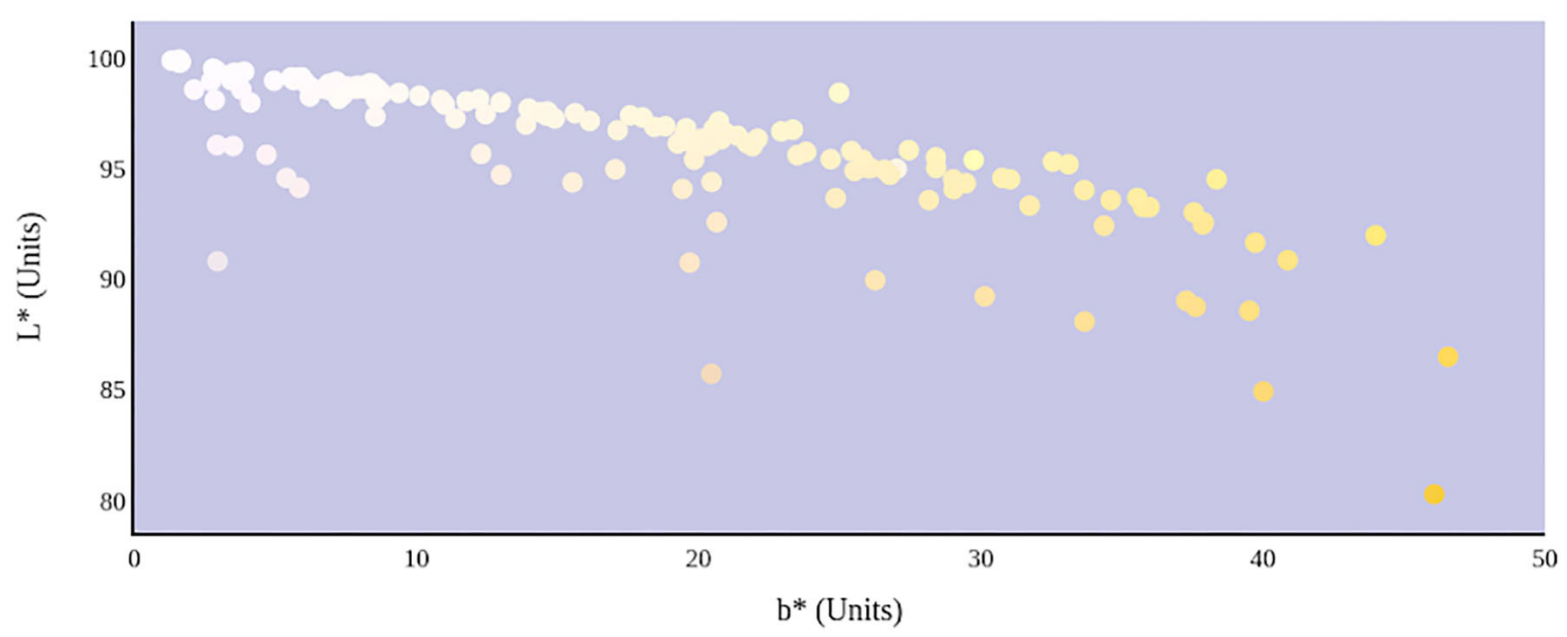
Hydration status significantly determines the lightness and yellowness of urine color in CIE L*a*b* color space.

**Figure 8:**
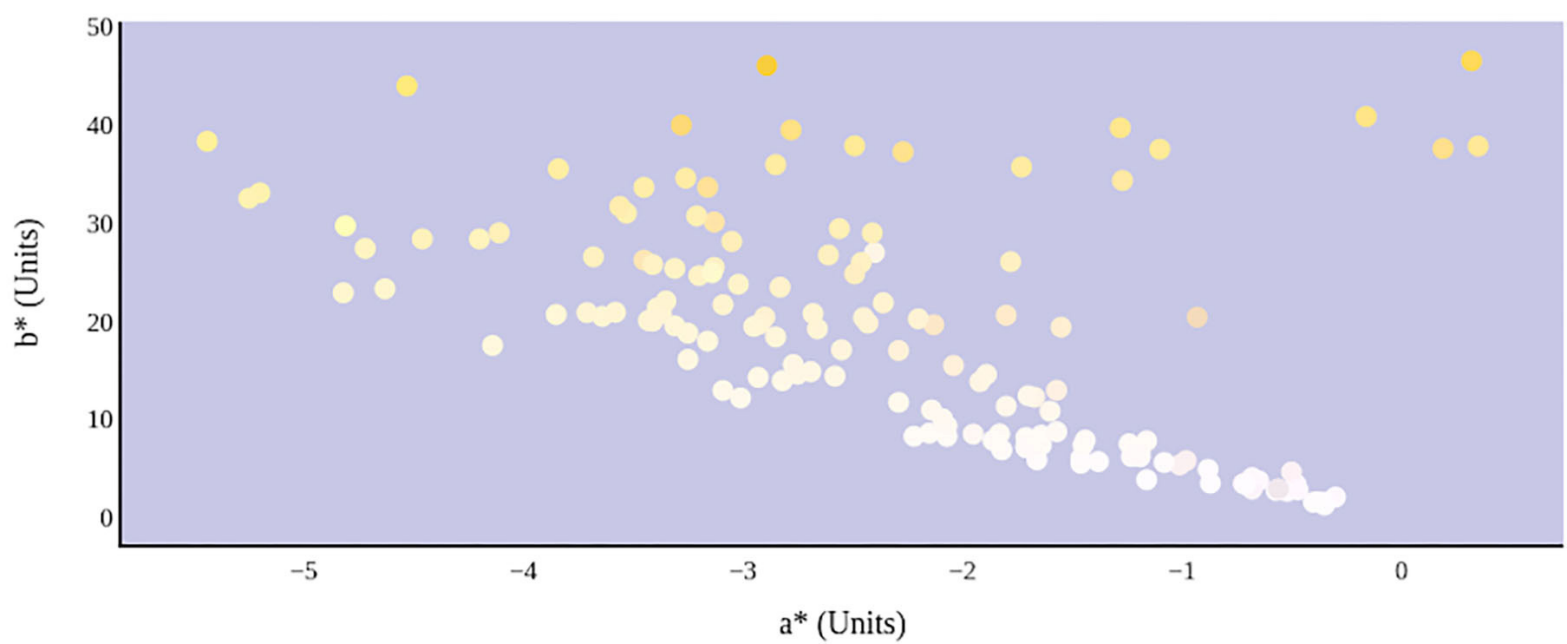
Hydration status significantly determines green–red and blue–yellow chromaticity of urine color in CIE L*a*b* color space.

The relationship between urine color and hydration status was visualized in the three-dimensional CIE L^*^a^*^b^*^ color space (Figure 6). An increase in dehydration resulted in darker and significantly more yellow urine, as L^*^ values decreased in lightness and b^*^ values increased positively along the blue–yellow axis (Figure 7). However, as dehydration increased, a notable polynomial trend in color along the green–red axis was observed as a^*^ values initially decreased negatively, indicating the presence of a green hue in slightly dehydrated urine, and then increased positively as urine became more concentrated and thus more dehydrated (Figure 8). Means and standard deviations for CIE L^*^a^*^b^*^ data are presented in Table 1.

Standardized beta coefficient values were then calculated for each of the three CIE L^*^a^*^b^*^ color parameters. This statistical analysis illustrated the relationship between each CIE L^*^a^*^b^*^ element and urine osmolality. It was determined that b^*^ was a significant predictor of urine osmolality (Beta = 0.836, *p* < 0.0001) and that a^*^ was a significant predictor of urine osmolality (Beta = −0.104, *p* < 0.029), whereas L^*^ was not found to be a significant predictor of urine osmolality (Beta = 0.006, *p* > 0.935) (Table 2). A hierarchal multiple regression model then was constructed to determine the predictive ability of all three CIE L^*^a^*^b^*^ variables for the criterion of urine osmolality. Based on the significance of each individual element's beta coefficient value, b^*^ was determined to be the most significant predictor of urine osmolality, followed by a^*^ and L^*^, respectively.

**Table 2:** Table of coefficients for a constructed hierarchal multiple regression model illustrating the predictive ability of CIE L*a*b* for urine osmolality.

**Model**	**Unstandardized coefficients**		**Standardized coefficients**	**Significance**
	**Beta**	**Standard error**	**Beta**	
Constant	74.702	634.485	–	0.906
L^*^	0.517	6.402	0.006	0.936
a^*^	−19.872	8.943	−0.104	0.028^*^
b^*^	19.947	1.834	0.836	0.000^*^

* *p < 0.05*.

The hierarchal multiple regression model was determined to be significant in its prediction of urine osmolality (*F*_(3,147)_ = 139.574, *p* < 0.0001, adjusted *R*^2^ = 0.735) (Tables 3, 4). Furthermore, it was determined that 74% of the variance seen in urine osmolality is due to the three predictor variables of CIE L^*^a^*^b^*^ color. The adjusted *R*^2^ value was used to determine this percentage of variance, as this value is based upon sample size and number of predictor values contributing to the regression model. The coefficients for each predictor value that contributed to the construction of the hierarchal multiple regression model have been reported (Table 2).

**Table 3:** Table of the analysis of variance for a constructed hierarchal multiple regression model illustrating the predictive ability of CIE L*a*b* for urine osmolality.

**Model**	**Sum of squares**	***df***	**Mean square**	***F*-statistic**	**Significance**
Regression^a^	9379312.37	3	3126437.46	139.574	0.000^*^
Residual	3292784.42	147	22399.894	–	–
Total	12672096.8	150	–	–	–

* p < 0.05.

a Predictors: constant, b*, a*, L*.

**Table 4:** Model summary for a constructed hierarchal multiple regression model illustrating the predictive ability of CIE L*a*b* for urine osmolality.

**Model**	***R***	***R*^**2**^**	**Adjusted *R*^**2**^**	**Standard error of the estimate**
Regression^a^	0.860	0.740	0.735	149.666

a Predictors: constant, b*, a*, L*.

## Discussion

Urine color analysis is an inexpensive and convenient method of identifying whole-body hydration status. This is important as monitoring of hydration is essential for the maintenance of essential physiological function and performance. Acute dehydration during physical activity can lead to impairments in cognitive and motor performance and increase feelings of tension, fatigue, and anxiety, and urine color analysis allows for a quick field assessment of an athlete's hydration status to estimate performance capacity (20, 21). To our knowledge, the only validated method of assessing urine color in a healthy adult population is with an established eight-point color chart that assigns a numerical value of hydration to an identified color (3, 4). Numerous studies using the subjective eight-point color scale have reported that an increase in subject dehydration results in darker urine color. For example, Armstrong et al. (4) reported that whole-body dehydration of −5.2% of body mass resulted in a significant increase in urine color from a mean value of 1 to a mean value of 7 on the eight-point scale. Simultaneously, urine osmolality significantly increased from 110 to 1,080 mmol/kg. However, this technique of color assessment is methodologically limited and, thus, subject to individual bias. The present study sought to identify the relationship between urine color and hydration status within CIE L^*^a^*^b^*^ parameters, a three-dimensional color space that incorporates three different color characteristics to present an objective visualization of color.

In this study, hydration status was determined by urine osmolality (1, 17). Kendall's Tau-b correlations revealed a significant relationship between urine osmolality and each individual CIE L^*^a^*^b^*^ color characteristic, with the strongest relationship seen between urine osmolality and b^*^ (Table 1). The b^*^ value is a chromaticity coordinate indicating a color's position along the blue–yellow axis. As dehydration increases, the concentration of yellow urochrome in urine increases as less water is being voided. This physiological response to dehydration results in both increased urine osmolality and yellow pigmentation of a sample, which supports the observed relationship between urine osmolality and b^*^. The significant relationship observed between urine osmolality and L^*^ is also expected. The L^*^ value is a measure of a color's lightness from white (100) to black (0). Urine samples following water consumption are dilute in color and will have higher L^*^ readings than samples collected following water deprivation which are more concentrated with urochrome solute.

The parabolic visual relationship and significant correlation between urine osmolality and a^*^ were not expected. The a^*^ value is a chromaticity coordinate indicating a color's position along the green–red axis. An increase in dehydration resulted in an initial decrease in a^*^, suggesting that the urine samples became greener. However, at a urine osmolality reading of ~600 mmol/kg, there is an observed increase in a^*^ values, suggesting that the urine samples became less green. Previous studies have proposed a urine osmolality threshold of <700 mmol/kg for indicating sufficient whole-body hydration (4, 17–19). The unexplained change in a^*^ occurring at this approximate threshold might be a result of physiological changes occurring in renal function as the body is preventing water loss. Alternately, it has been suggested that pH of the surrounding medium may alter the absorptive spectra of linear tetrapyrroles like urochrome (22). Thus, the possibility exists that urine pH and urine osmolality may also exhibit a parabolic relationship. Certainly, further work is warranted in the area.

The successful findings in the correlation analysis prompted the construction of a regression model that suggests predictive ability of CIE L^*^a^*^b^*^ for urine osmolality. This model determined that 74% of the variance seen in the urine osmolality of the collected samples was due to the predictor variables of CIE L^*^a^*^b^*^ color (Table 4). This is a powerful finding, as it further supports the strong relationship observed between these two measures of urinalysis.

The relationship between urine color and hydration status in CIE L^*^a^*^b^*^ color space was visualized in a three-dimensional model (Figure 6). CIE L^*^a^*^b^*^ provided an objective analysis of urine color, in which a quantitative change in any or all of the three-color characteristics resulted in the same change in visual color perception. Therefore, assessment of urine color through CIE L^*^a^*^b^*^ provides a more complete understanding of the relationship between urine color and hydration status, such as the unexpected movement of a^*^ along the green–red axis. Such nuances and shifts in tone cannot be identified with a subjective color chart. The color chart has also shown to be methodologically limited (8–10) as well as inconclusive for measuring hydration status in older populations (11, 12).

Limitations of the current study include that urine osmolality was used as the sole marker of hydration status. Currently, there is great debate in the literature as to what is the best marker of hydration status, including laboratory measures such as plasma osmolality, urine-specific gravity, and urine osmolality. However, past studies have successfully used urine osmolality as a surrogate of hydration status (1, 17). Furthermore, diet and vitamin intake were not controlled for in the current study, and these factors have the potential to artificially alter urine color and urine osmolality. It should also be noted that this dataset was a time series, as each subject contributed multiple samples over the course of the collection period, and thus, multiple data points were obtained from each subject. This violated the assumption of independent data and was recognized during statistical analysis.

This present study sought to understand and quantify the relationship between urine color and hydration status when evaluated in CIE L^*^a^*^b^*^ parameters. It was discovered that significant relationships exist with urine osmolality and each CIE L^*^a^*^b^*^ color characteristic. A hierarchal multiple regression model for predicting urine osmolality from a color's position in the CIE L^*^a^*^b^*^ color space was also presented. This suggests a potential method of determining one's hydration status using objective color assessment. To our knowledge, this study is one of the first to evaluate the use of objective color measurement to assess hydration. Further research should examine the relationships between objective color assessment and other methods of urinalysis that are used to dictate hydration status.

## Data Availability Statement

The raw data supporting the conclusions of this article will be made available by the authors, without undue reservation.

## Ethics Statement

The studies involving human participants were reviewed and approved by San Diego State University Human Research Protection Program. The patients/participants provided their written informed consent to participate in this study.

## Author Contributions

MB conceived and designed the research study. MB, RB, TE, AM, and VR performed the measurements and collected all necessary data. MB and RB performed the statistical analyses and wrote the manuscript in consultation with TE, AM, and VR. The full dataset is available upon request to RB. All authors agree to be accountable for the contents of the manuscript.

## Conflict of Interest

The authors declare that the research was conducted in the absence of any commercial or financial relationships that could be construed as a potential conflict of interest.
